# Advance on the biology, behaviour ecology and management of the coffee white stem borer, *Xylotrechus quadripes* Chevrolat, 1863 (Coleoptera: Cerambycidae)

**DOI:** 10.1016/j.heliyon.2023.e19506

**Published:** 2023-08-29

**Authors:** B. Kariyanna, Zhicheng Xiong, Nidagundi Pradnyarani, Sunil Kumaraswamy, G. Ramkumar, B.V. Subba Reddy, Xinnian Zeng

**Affiliations:** a Fluoro-Agrochemicals, CSIR-Indian Institute of Chemical Technology, Tarnaka, Hyderabad, India; b College of Plant Protection, South China Agricultural University, Guangzhou, Guangdong Province, China; c University of Horticultural Sciences, Bagalkot, India; d Department of Agricultural Entomology, University of Agricultural Sciences, GKVK, Bangalore, India; e Department of Entomology, University of Georgia, Griffin 30223, GA, USA

**Keywords:** Coffee white stem borer, Biology, Host selection, Ecology, Management

## Abstract

The coffee white stem borer, *Xylotrechus quadripes* Chevrolat, 1863 (Coleoptera: Cerambycidae) – here removed from the synonymy with *X. javanicus* (Laporte & Gory, 1841) *–* is the most notorious pest in Arabica coffee plantations in many southeast Asian countries. It can cause damage up to 80% in various gardens. The borer is reported on 16 different host plants other than coffee. The severity of the pest was more commonly recorded on the Arabica coffee than on other species. More pest intensity on the coffee may be due to its innate evolutionary relation compared to other host plants. Studies revealed that the borer is more specific and attracted to the volatile of coffee plants but it is still needs a strong supporting data. Some of the behavioural and ecological-adaptations of borers leads to avoid predation and chemical-pesticides reaching them. Hence, no single method gives perfect control of this pest; therefore, harmonic use of different tools such as cultural, mechanical, physical, bio-control and chemical methods are the best way to combat this pest. Though the pest is economically important, the information on chemical and ecological behaviour, host plant resistance and recent advancements in the pest management are scanty. The present article is an endeavour to shed a light on biology, behaviour, host selection and management of *X. quadripes* with multiple instances, that will give a new avenue for the researchers to work on the least concerned fields to develop strong management practice and alert against future pest outbreak.

## 1 Introduction

**Fig. 1:**
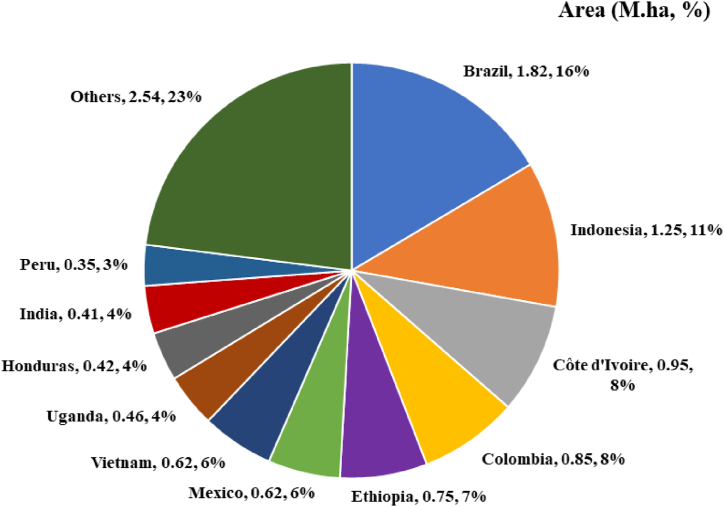
Major coffee growing countries across the globe (Data source: https://www.atlasbig.com).

The commercial plantation crop coffee is widely grown across the globe (Fig. 1). By cultivating *Coffea arabica* L. and *C. canephora* Pierre (synonym: *C. robusta*) India stand one among the largest producer in the world and exporting 395,000 tonnes annually [1,2]. Typically, Indian farmers grow coffee under shade trees and the rest of the world cultivated as monocrop [3]. India earns 836 million $ through export [4]. Nearly 56% expansion happened in past 25 years in the area of coffee plantations including different parts of the Western Ghats [2,5]. Indirectly, the coffee plantations created special conditions for wildlife conservation but along with that, it also harbour the important pest called coffee white stem borer (CWSB), *Xylotrechus quadripes* [6]. The species such as *X. quadripes* in South-East Asia (including India and China) and the African coffee white stem borer, *Monochamus leuconotus* (Pascoe, 1869) (Coleoptera: Cerambycidae) in Africa are more destructive on Arabica coffee [[7], [8], [9], [10]].

**Fig. 2:**
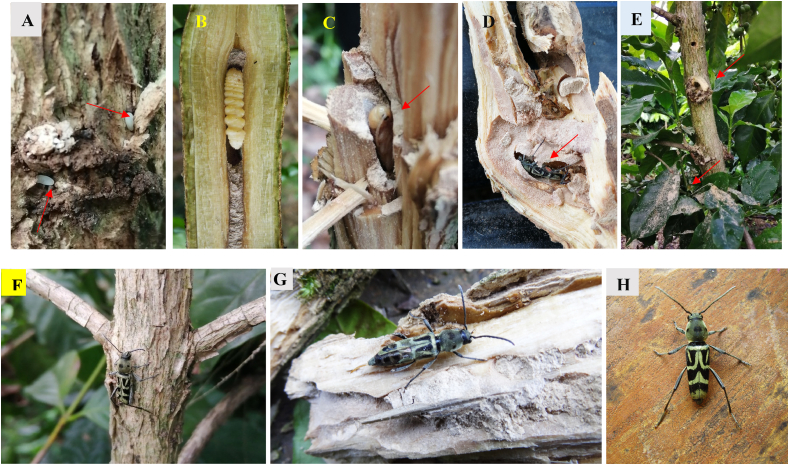
Boring activity of the coffee white stem borer on the stem, A: egg laid on the bark, B: larva inside the stem C: pupa inside the stem D: adults inside the bored stem, E: stem with holes and powdery dust on the leaves caused by borer, F,G,H: adult spotted on the different location.

Larvae of *X. quadripes* bore tunnel into the main stem and lateral branches affecting the vascular transport system causes wilt and in severe cases leads to death of plants. *C. arabica* is the most susceptible species compared to *C*. *canephora*. In favourable conditions, larvae bore the stem for 1–1.5 years inside and adults emerge by making exit after complete development (Fig. 2) [7,[11], [12], [13]]. Generally, there are three to four pest generation peaks observed and the emergence of adults purely depends on climatic conditions [14]. This pest got greater attention after the banning of dieldrin insecticide and neglected coffee plantation. Due to borers internal feeding nature, it is difficult to manage this pest and only few effective chemicals are available till date. Recently, another important practice such as host plant resistance became an important alternative to insecticides [12] followed by biological control *viz.,* parasitoids, predators and entomopathogens [15] and trapping by pheromone [16] are using in management of the CWSB. Management of this pest is troublesome and researchers tried several strategies to reduce the pest load in plantation but it was not effective. That may be due to wrong identification, so use of morphological data with molecular diagnostic tool may be effective [17]. Further, bio-ecological study and inter-relationship with given agro-ecosystem of the CWSB in Asian and Indian regions support its management through sustainable way [9,10,12,18,17], because to implement the integrated pest management technique complete information on stem borers is the prime need. The article is designed by collecting the information related to systematics, biology, chemical and behavioural ecology and management. The information related to taxonomic and distribution chao has been answered by referring the most appropriate data. The discussion related to species status, behavioural ecology and tolerant varieties are new components compared to previous reviews. We also discussed scantly on the areas of biology, host selection and new techniques to manage the CWSB in a different agro-ecosystem, that will aid the researchers to think on the burning issues to formulate new pest management technique.

## 2 Taxonomy, origin and pest status

*X. quadripes* belongs to order Coleoptera, family Cerambycidae, subfamily Cerambycinae and tribe Clytini. Dauber and Hawkeswood [19] synonymised it with *X. javanicus* (Laporte & Gory, 1841), a species widespread in Malayan peninsula, Singapore, Borneo, Sumatra and Java. However, *X. javanicus* considerably differs from *X. quadripes* in the pronotal pubescence (a basal and two lateral hairless spots, sometimes connected to form a T-shaped spot in *X. javanicus vs.* three well separated median spots in *X. quadripes*), the shape of the postmedian elytral band (almost narrow in *X. javanicus vs.* advanced anteriorly in *X. quadripes*) and the colour of femora (always brownish black in *X. javanicus vs.* sometimes red in *X. quadripes).* Consequently, *X. quadripes* is removed here from the synonymy with *X. javanicus.* The only synonym of this species is *Cucujus coffeophagus* Richter, 1867; *Xylotrechus lyratus* Pascoe, 1869 and *Clytus sappho* Pascoe, 1858 are synonyms of *X. javanicus.*

**Fig. 3:**
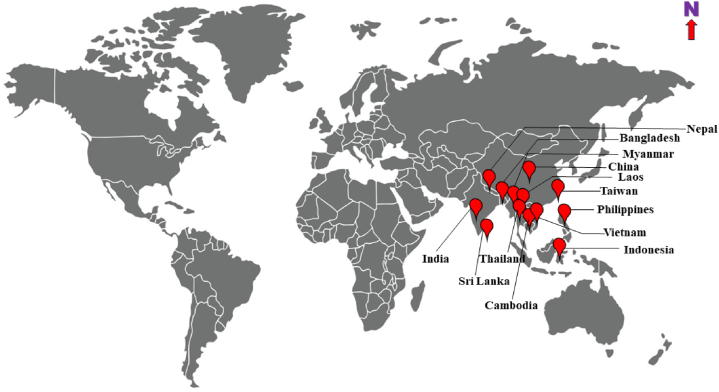
Distribution of the *Xylotrechus quadripes* on the coffee ecosystem across the world.

*X.* q*uadripes* was first recorded in India in 1838 [14,20] and it distributed in various Arabica coffee growing tracks *viz.,* Karnataka, Kerala, Tamil Nadu and Andhra Pradesh [21,22]. This pest is also recorded from Myanmar, China, Java, Nepal, Sri Lanka, Thailand, Bangladesh and Vietnam (Fig. 3) [20,[22], [23], [24], [25], [26], [27], [28], [29], [30], [31]]. One article [10] has published the presence of *X. quadripes* in Africa, North and South America but no proper supporting evidences.

*X. quadripes* was not recorded in South Africa [32], but Schoeman [33] erroneously quoted the African coffee white stemborer, *Monochamus leuconotus* as *X. quadripes* and this wrong identification was referred by many authors *viz.,* Venkatesha and Seetharama [34] and Jayarama et al. [35]. In India, *X.* *quadripes* can cause a yield loss of 17.8–20% and in Nepal the loss was up to 60% [36,37]. The infestation was more prominent in highland (9.7%) area compared to lowland (6.6%); similarly old crop (8.3%) is more prone to CWSB compared to young one (0.7%) [38]. But a recent report stated that it was severe (20% loss) in low elevation and lesser rainfall area [39]. This pest causes around 17–40 million $ crop damage yearly [6].

### 2.1 Host plants of the stem borer

Though this pest attacks on two species of Coffee, *Coffea arabica* is the primary and most preferred host by stem borer and occasionally it attacks weak *C. canephora* and *C. liberica* plants (Table 1) [40]. *C. canephora* having, smooth bark, thicker primary and harder branches, makes them not suitable for growth and development of CWSB. Due to smooth and thick bark, females do not prefer to lay eggs because it exposes larvae to predators and parasitoids [41,42]. Other than Arabica and *C. canephora*, this pest occurs in Asia on other alternative wild host plants, *viz., Canarium* sp., *Cudrania javanensis, Gardenia* spp., *Ixora coccinea*, *Jasminum dispermum*, *Olea dioica*, *Oroxylumn indicum*, *Premna pyramidata*, *Psilanthus bengalensis*, *Pterocarpus marsupium*, *Trema orientalis*, *Randia dumetorum*, *Randia spinosa*, *Rhus semiciliata, Tectona grandis* and *Wendlandia myriantha* (Table 1) [14,30,[41], [42], [43], [44]], but no serious considerable damage on these host species was recorded [16]. The CWSB occasionally breed on standing tree of *O. dioica* and dry logs of *T. grandis* [14,40] and cut woods [43] but they will not thrive in large number. Importantly, the CWSB attracts more towards the plants adhered with larval frass and coffee sawdust [45]. Many host species of CWSB belong to Rubiaceae and later they evolved to adopt more specific for Arabica species in Asian countries [14]. Leaving the debris and saw dust after pruning invites the CWSB, so maintaining clean garden is advisable. Avoiding the above-mentioned hosts as a shade crops, ornamental or for woody purpose around the coffee plantation helps to check the borer activity during no crop time.

**Table 1:** Important host plants for *Xylotrechus quadripes*.

Intensity of damage	Family	Species
Major	Rubiaceae	*Coffea arabica* L.
Minor		*C. robusta* Linden*, C. liberica* Hiern*, Ixora coccinea* L., *Gardenia* spp., *Psilanthus bengalensis* Lero, *Wendlandia myriantha* How, *Randia dumetorum* L., *Randia spinosa* (Thunb.)
	Burseraceae	*Canarium* sp.
	Moraceae	*Cudrania javanensis* Trecul, *Antiaris toxicaria* Lesch
	Oleaceae	*Jasminum dispermum* Wallich, *Olea dioica* Roxb.
	Bignoniaceae	*Oroxylumn indicum* L.
	Lamiaceae	*Premna pyramidata* Wall,
	Fabaceae	*Pterocarpus marsupium* Roxburgh, *Albizzia* sp.
	Cannabaceae	*Trema orientalis* L.
	Anacardiaceae	*Rhus semiciliata* L., *Lannea coromandelica* (Houtt.) Merr.
	Lamiaceae	*Tectona grandis* L.

### 2.2 Pest status

In around 70 countries across the world, the production of *C. arabica* is an important economic activity with $20 billion annual trade and millions were employed in the complete value chain [[46], [47], [48]]. Insects are the major constraint in achieving higher productivity, though the production is surplus. There are more than 100 insect species that have been reported on coffee as a pest [20]. Among these pests the CWSB was considered as the most severe on coffee plants causing crop loss up to 26–40 million $ [[49], [50]]. Chronologically, the annual loss caused by CWSB in India was 0.64 million US$ in 1987 and 1997 [50,51] but it increased during 2006 to 40 million US $ [49] and later in 2010 it reached to 26 million (Table 2) [52]. This pest started causing serious damage to coffee plants in mainland of China since late 1950’s, as the expansion of plantation in larger-scale and the pest also caused 47% damage to *W. myriantha* in a mountainous area [53]. Recently, in an established Arabica coffee plantation of Tamil Nadu, 17.7% loss was recorded by CWSB [54]. The increased losses are due to the expanded area of production of the coffee across South and South-East Asia.

**Table 2:** *Xylotrechus quadripes* distribution and its damage status across the world.

Country	Economic damage (%)	Reference
India	40 or $26–40 million	[49,55]
China	39–42	[56]
Nepal	30–70	[57,58]
Thailand, Sri-Lanka, Vietnam, Myanmar, Java and Indonesia	Destructive pest with no data on annual economic damage	

## 3 Biology and life cycle

Due to boring nature of the larvae and long adult life cycle with good flying capacity, the biology of CWSB looks typical. Pre-oviposition period of adults is 2 days and around 9 days are the oviposition period [20,27]. Females lay eggs in dark conditions [59]. The detailed biology, morphometry and life cycle of the all the developmental stages of CWSB are presented below.

### 3.1 Egg

**Fig. 4:**
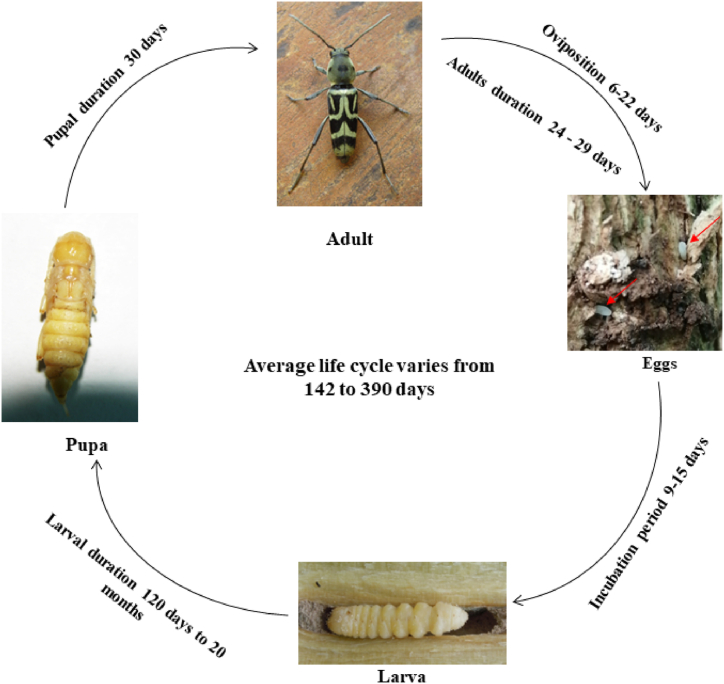
The complete life cycle of the *Xylotrechus quadripes*.

Females lay about 50–100 eggs in bark cracks of the main stem rather than primary branches of coffee plants during bright sunlight from 12.00 to 16.00 h. The oviposition period range from 6 to 22 days (Fig. 4) [60,61]. The eggs are milky white in colour with an oval shape at the initial stage and later turn into yellowish colour. The size of the eggs varies up to 1.25–1.28 mm long and 0.46–0.5 mm wide [[20], [27], [62], [63], [66]]. Up to 103 single eggs were laid by a female, but occasionally it lay in the group which ranges from 1 to 10 [27,62]. Among the total laid eggs of stem borer, 78% hatch into larvae in 9–15 days, later in the first and second instar larvae mortality are high [27].

### 3.2 Larva

The larva is eucephalous with strong mandibles. Fully grown larva is apodous with dark brown head capsule and yellowish body contain cylindrical segments. The size varies from 7 to 25 mm in length and from 2.3 to 5 mm in width [20,27]. The larva completes five instars in India whereas six instars in Thailand [27,63]. The larva feed on the core part of the lower stem, chew the bark and make tunnel into the stem where they end up obstructing the flow of food, which may even kill the plant. Plants attacked by CWSB turns yellowish with rings on bark and the presence of an exit hole with frass are the major symptoms. The affected plants appear reduced vigour, wilted, stunted growth with the sign of dieback and reduced fruit set [16,64]. Larva can survive inside the bored hole from 120 days to 20 months in standing crop ((Fig. 4) [63,64,65] and 70 days in dry stems [27,63]. Fully grown grubs make a tunnel towards the stem and a small exit hole that helps the adult emergence [20,27].

### 3.3 Pupa

The pupa is exarate with free appendages and yellow, its length varies from 12 to 19 mm and width of 2.3–6 mm [20,27]. The pupal stages last for 30 days on a standing plant (Fig. 4) but on dead logs or dry stem it completes in 9 days [20,27,66,67]. Before adult emergence, they remain in the pupal case for 3–7 days [20,27].

### 3.4 Adult

**Fig. 5:**
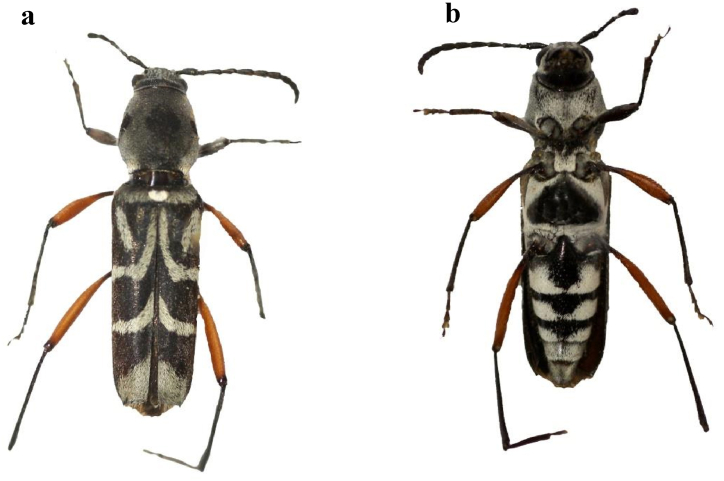
The female of *Xylotrechus quadripes* a: dorsal and b: ventral view (Source: Kariyanna et al.*,* [66]).

Adults of *X. quadripes* are elongated and slender. The head, pronotum and elytra covered with greyish white pubescence [[66], [64], [65]]. Head of both male and female sex have different raised structures [23]. The adult of male and female are easily differentiated by abdominal tip *i.e* the female abdominal last segment is long and tapered with semi-circular, but the male has broad, short and rectangular tip (Fig. 5). The average length of a female is 13.5 mm whereas male measures 11.4 mm [31,34,63]. Adult males are more active than compared to females [32]. The adults can damage on buds, shoot and bark of the stem that leads to reduced productivity and production (Fig. 2) [65]. Based on the size and age of the plant the life cycle lasts between 142 and 390 days (Fig. 4) [37,43,63]. In India under normal field conditions, it takes one year to complete its life cycle [40]. Generally, only one generation per year is observed in India [27], but in China, there are two generations recorded [68]. A study by Visitpanich [27], indicated that in India also this pest complete two generations. This type of difference may be influenced by the environmental conditions and place of the host plant [14]. The sex ratio is 1:1.22 (female/male) in India [62], whereas 1.63:1 is recorded in Thailand on dry stems [27], maybe because of the availability of nutrient and environmental conditions. The adult mean longevity varies from 24 to 29 days for males and females respectively [27,69].

## 4 Adult behaviour

The adult males are more active fliers and travel long distance [13,59,70]. The pre-monsoon (April to May) to the post-monsoon period (October to November) is the window for the adult emergence from the bored hole [21]. An average of 25 seconds required for adults to start mating after the emergence [61]. The adults mates multiple time (5.07 ± 1.49) with same male at 9:00 and 12:00 h and 15:00 and 17:00 h to continue next generations [61,71,72]. Pheromones are the primary source for the intercourse of females and males [14,45], but synthetic pheromone used for management of borer, failed to attract a significant number of adults beetles [28,49]. Both male and female produce the pheromone; male produces the long range and female produce for short range, but the maximum success rate of mating is achieved in female pheromone compared to male [43]. Previous studies indicated that beetles mate on the different parts of coffee plants and it does not use visual cues for mate selection [71,72]; on the other hand, Venkatesha and Dinesh [14] reported that the borer was attracted towards coffee plants by volatile compounds. Based on the preference report towards certain host plants by volatile compounds, there is a great chance to develop attractants or non-host repellents [6]. The chemoreceptor genes repertoires such as odorant, gustatory, ionotropic and sensory neuron membrane proteins are the main governing agents for adult mating, host seeking and recognition of *X. quadripes* [73]. Advance study in respect to the proteins and their governing gene helps to understand the detailed behavioural adoption of the adults.

## 5 Ecological adoption

The CWSB is having strong innate relation with coffee varieties and adopted several ways to escape from predators. Adults lay the eggs under the bark to avoid the reach of natural enemies [34]. The grubs make a bored holes inside the stem, powdery masses formed by cutting it and later feed on the deposited powdery mass. The grubs fecal pellets and frass filled in the tunnel, such packed tunnel protects the grubs from natural enemies [27,74]. Emerged adults usually mate and deposit eggs on the same day or in the next of the emergence [20,71]. Studies revealed that antennae are involved in courtship and mating, but the vision plays no role and mating takes place during 9 a.m.–12 noon and between 3 p.m. and 5 p.m. There is a controversy between the attraction of individuals for courtship: Veeresh [43] confirmed that males are attracted by females, but on the contrary, Venkatesh et al. [43] reported females are attracted by males. This behaviour is linked to an evolutionary connection for one sex to attract others [75]. A study says, mating typically occurs before host selection and host plants may not be the obligate site for CWSB mating, with only 1% of mating occurring on coffee plants and the majority of mating occurring in other locations such as cage nets (53% mating) and release sites (46% mating) [6], where both sexes find each other via pheromones [43,71]. Study and identify the right compound from the either of the sex and preparing the strong blend will helps to mass trap adults in large scale. Use of sex pheromone with proper blend act as an additional tool for monitoring and mass trapping of the male CWSB.

### 5.1 Host selection

Although CWSB has been reported to cause damage to coffee plants in open field conditions (tropic and subtropic) and in temperate regions, the research on the host selection behaviour in shaded rainforest conditions of Indian coffee plantations is limited. Murphy et al. [16] and Prashant [76] confirmed the beetle affinity for the host plant by presenting green tissues, stems and branches of coffee, but only a few borers were attracted to the host plant. Rajus et al. [6], reported that, the beetles approach coffee plants by flight, indicating that beetles perceive cues from the host plant while flying and typically land on coffee leaves. Further, the choice assays in Arabica coffee using different combinations of treatments such as Arabica plant with leaves, no leaves, infested, or low leaf rust, among these treatments 78% beetles preferred coffee plants with leaves over no leaves, indicating the role of leaf volatiles in the host plant selection [6]. It implies that the use of the plant volatile as food bait and sex pheromone (attractant) for developing the pheromone, act synergistically for mass trapping of the both male and female CWSB effectively.

### 5.2 Role of plant volatiles in host selection

The antennal transcriptome of the *X. quadripes* contains uridine diphosphate (UDP)-glycosyltransferases (UGTs) enzyme, which potentially acts as odour perceiving agent from the different sources [77]. Olfactory and visual cues of coffee plant could serve as important host selection guides for CWSB. The volatile compounds *viz.,* (+)-limonene, β-caryophyllene, β-pinene, styrene, (−)-α-muurolene, (−)-α-cubebene released from different host plants act as cues for host selection by CWSB [78,79]. Rajus et al. [6] conducted an experiment by covering the coffee plant with transparent ventilated plastic sheet and non-ventilated transparent plastic sheet. The results displayed that, about 78% of the beetles uses volatile cues to locate host by choosing the coffee plant kept in ventilated transparent sheet compared to plant covered with transparent non ventilated plastic sheet.

The (E)-2-hexenal, (Z)-3-hexenol, (E)-2-hexenol, ethyl benzoate, methyl salicylate and indole were identified as plant volatiles from coffee bark. Arabica coffee bark and branch contained more volatiles than *C. canephora* coffee bark and branch, which could explain CWSB's preference for Arabica coffee [76]. Furthermore, Rajus et al. [6], identified several volatile compounds in Arabica and *C. canephora* using solid-phase microextraction (SPME) and discovered few similar compounds such as (E)-2-hexenal, (Z)-3-hexen-1-ol, (E)-2-hexen-1-ol, (±)-linalool and methyl salicylate in both Arabica and *C. canephora*. GC-EAD experiments confirmed that the beetles responded to 12 identified volatile compounds, such as (Z)-3-hexen-1-ol, acetophenone, (E)-2-hexen-1-ol, (E)-2-hexenal, n-dodecane, methyl salicylate, tetradec-1-ene, benzyl alcohol, 2,6,11-trimethyldodecane, caffeine, 5-ethyl-2,5- dihydrofuran-2-one and (E)-3-hexanoic acid (Table 3), indicating the role of these compounds in host selection. Similarly, terpenes (monoterpenes and sesquiterpenes), esters, alcohols, and ketones were found in the volatile components of four *X. quadripes* host plants: *C*. *arabica, Artocarpus heterophyllus, Alstonia scholaris* and *Psidium guajava.* The important common volatiles of four plants, such as (+)-limonene, styrene, β-pinene, (−)-α-muurolene, (−)-α-cubebene and β-caryophyllene which could serve as cues in host plant selection [78,79].

**Table 3:** Response to plant volatile compound by male and female of *Xylotrechus quadreipes*.

Sl. No.	Name of the plant volatile compound	Male	Female	Remarks
1	(Z)-3-hexen-1-ol			
2	Acetophenone		NS	
3	(E)-2-hexen-1-ol		NS	
4	(E)-2-hexenal	NS		
5	n-dodecane	NS	NS	
6	Methyl salicylate	NS	NS	
7	Tetradec-1-ene	NS	NS	
8	Benzyl alcohol		NS	
9	2,6,11-trimethyldodecane	NS	NS	
10	Caffeine	NS	NS	
11	5-ethyl-2,5- dihydrofuran-2-one	NS	NS	
12	(E)-3-hexanoic acid	NS	NS	
13	(±)-linalool			Potential compounds for food baits
14	(±)-α-pinene			
15	(−)-β-pinene		NS	
16	(E)-β-caryophyllene	NS	NS	
17	(R)-(+)-limonene	NS		
18	(E)-β-ocimene	NS	NS	

: Indicate significant response by the adults to volatile compound; NS: No significant response. The data fetched from Rajus et al. [6]; Luo et al. [79].

In the EAG assay, male CWSB antennae responded significantly to (E)-2-hexenol, benzyl alcohol, (±)-linalool, acetophenone, (Z)-3-hexenol, (±)-α-pinene, (−)-β-pinene and (E)-β-caryophyllene, but there was no significant response to methyl salicylate, 1-nonanal, n-dodecane, γ-terpinene, (R)-(+)-limonene and tetradec-1-ene. Similarly, the female CWSB antennae significantly responded to seven compounds *viz.,* (Z)-3-hexenol, (±)-linalool, (±)-α-pinene, (R)-(+)-limonene, (E)-β-ocimene and (E)-2-hexenal (Table 3) [6,78]. This suggests that volatiles from the host plant stimulate the olfactory sensilla of CWSB, eliciting a response and directing them to the coffee plant. The volatiles and pheromones may act as a best combination to catch the adults of CWSB.

When male and female beetles were exposed to a mixture of male pheromone and plant volatiles in various ratios in a wind tunnel, field wind tunnel, EAG and field trial, the highest response was observed for (1:1) ratios of the compounds such as (E)-2-hexenal: 2-hydroxy-3-decanone (1:1), followed by (Z)-3-hexenol: 2-hydroxy-3-decanone (1:1) [76]. Similarly, Rajus et al. [6] tested the identified volatile blends in comparison with the pheromone lures in coffee ecosystem. CWSB attracted equally towards active volatile blends and pheromone, but the number of trap catches were low. So, still there is wider scope in the area of chemical ecology to use plant volatile as food lure and pheromone as sex lure for effective catching of the adults.

## 6 Management

The CWSB management aspect has seen significant success after ten decades of consistent effort but it is not uniform across the coffee growing area due to local climate, natural enemies, grower consciousness, elevation and many other factors. Understanding the pests behavioural ecology is critical because it serves as a base for pest management either naturally or artificially. To manage this pest, comprehensive pest management practices such as resistant/tolerant cultivars, cultural, mechanical, physical, biological and chemical methods have been developed.

### 6.1 Tolerant genotypes

*C. canephora* coffee plants were unaffected by CWSB in Kodugu districts of Karnataka [80]. Further, the S.4595 cultivar (crossed with Sln.11 x HDT) reported to tolerant against CWSB from Chikkamagaluru district of Karnataka [81]. Recent study from Pokhara, Nepal, reported that the Arabica cultivars like Amarillo group, Catimor, Indo Tim-Tim, Ketisic, Pacas, San Ramon, Syangja special and both Vermelo group showed no infestation by CWSB [82]. Importantly, *CYP79* gene of *C. canephora* involved in various pathways that produce defence compounds *viz.,* cyanogenic glucosides, glucosinolates, and herbivore-induced volatile compounds [83]. *CYP79* gene can be used as a candidate gene for developing resistant varieties by understanding its molecular structure. Further, the development of the resistant varieties required starting materials for the study. The above listed varieties can be used for developing a CWSB resistant variety with high yielding ability.

### 6.2 Cultural

This method combines with agronomic practises such as crop management to facilitate a suitable crop growth environment and reduce borer activities. Cultural practices such as shade maintenance, tracing, uprooting and disposal of infested stems prior to CWSB flight periods may reduce borer severity [84]. Proper shade management, optimal soil and moisture conditions helps to reduce infestation levels. Improved shade management can be achieved by planting shade trees 1–2 years ahead of the coffee plants [85].

In order to achieve the lower infestation of the CWSB, reduce the exposure of the bark or limiting the female access to the bark. Softening the bark of the coffee stem with maize cobs above 0.5 m soil reduces the female preference for oviposition on the softened bark [86]. Covering the bark with banana leaves, gunny bag and non-woven cloth materials prevents female oviposition [86,87]. Between March and September, the scaly bark is covered with thick materials such as jute sacks, gunny bags or rough cloth and pasted with a mixture of red soil and cow dung to prevent borer settling and egg-laying [87].

The summer flight period of CWSB typically begins in April, so to effectively manage the CWSB population, avoid shade during the months of March and April. The shade regulation can be carried out at the end of May or in June [88,89]. Plants with low levels of pest infestation or suspected of being infested can be saved by wrapping the main stem and thick primaries with strips of non-woven fabric material (1 mm thickness) by the end of March. Wrapping prevents adult beetles from emerging from infested plants, preventing the infestation from spreading further. If the infestation is in its early stages, the wrapped plants will also recover [89].

### 6.3 Mechanical

To avoid increased pest load, effective CWSB management requires some mechanical approaches such as handpicking the pest in the early stages of incidence, removal and destruction of infested plants before adult emergence in March and September. Scrubbing the main stem in open conditions reduces pest infestations because removal of loose bark hinders female egg laying and it can be recommended with coir gloves due to its safe and efficient nature [14,85]. Use of wire-spoke can effectively kill the grub inside the boring tunnels [86]. Observing and killing adults would be a very effective method but it is labour-intensive process that would be difficult to implement in large plantations.

### 6.4 Behavioural or physical

Mating behaviour of CWSB relies on pheromone communication and 2-hydroxy-3-octanone, 2,3-decanedione, 2-phenylethanol and octanoic acid produced by male CWSB was found to elicit antennal responses in both male and female CWSB. However, 2-hydroxy-3-decanone as a trap bait was not successful in managing CWSB populations in China due to the complex mating behaviour [45,49]. Other sex pheromone components *viz.,* 3-hydroxy-2-decanone and 2S,3S-dihydroxyoctane and four other compounds such as, 2-hydroxy-3-octanone, 2-phenylethanol, octanoic acid and 2,3-decanedione have been identified from male volatiles [45]. The female aggregation pheromone, 1-octadecene and male pheromone, 2-hydroxy-3-decanone (2H3D) at 1:2 ratio attracted more beetles and males use 1-octadecene for mate finding and can be utilized for monitoring and mass trapping [90]. The use of a cross-vane trap with a pheromone compound containing 2-hydroxy-3-decanone captures more female borers during flight time [85,87].

In the coffee plantation, yearly twice (April–May and October–December) the cross vane pheromone traps are used to attract the beetles during the peak flight periods [6]. The trap can be placed at a height of 6–6.5 feet from the ground and placed at every 8 to 10 plant or 20 m distance in grid [91]. Install pheromone traps at a rate of 10 traps per acre before the first two weeks of April to attract female beetles. Trapping can be effective in bringing down the pest incidence and also helps in proper monitoring of the pest in the field [14]. Other than pheromone traps, light trap also can be used but light traps are not effective like pheromone traps to catch the beetles [58]. Further, covering the main stem with polythene and wire mesh or strips of date palms can greatly reduce pest load, but it is a time-consuming and inefficient process. Wrapping the stem in non-woven fabric, followed by an insecticide spray, resulted in 100% pest mortality. Wrapping and covering must be need based during high beetle activity period, which was discussed in cultural practice.

### 6.5 Biocontrol

The coffee ecosystem is very diverse, but the internal feeding nature of the CWSB limits access to natural enemies. Many researchers recorded diverse biocontrol agents in coffee ecosystem that helps to keep the population of CWSB under control to some extent (Table 4). The exposure of the borer to natural enemies is increased by proper shade management to maintain sunlight. Borers main enemies in the gallery are woodpeckers, green barbet and ants, so creating a conducive environment for their coexistence aids in borer management [70]. Microbial agents that attack the borer include *Beauveria bassiana* and *Metarhizium anisopliae*, which are most host-specific [37,70]. Potential microorganisms such as *Aspergillus* spp., *Cladosporium* spp., *Fusarium* spp., *Penicillium* spp., few bacteria and yeast could be used to manage the borers because they posed a significant threat in laboratory artificial diets of CWSB [92].

**Table 4:** Different natural enemies recorded on the CWSB from Asian countries.

Sl. No	Family	Species	Reference
Hymenoptera Parasitoids			
1	Aulacidae	*Pristaulacus* spp., *Pristaulacus nigripes* Kieffer	[20,27]
2	Bethylidae	*Apenesia* sp., *Apenesia sahyadrica* Azevedo & Waichert, *Mysepyris grandiceps* Kieffer, *Sclerodermus domesticus* (Latreille), *Sclerodermus* sp. and *Sclerodermus vigilans* Westwood	[[38], [93], [94], [95]]
3	Braconidae	*Campyloneurus* sp., *Dorcyctes bistriatus* Kieffer, *D. brevipetiolus* Kieffer, *D. compactus* Kieffer, *D. coxalis* (Szépligeti), *D. picticeps* Kieffer, *D. stroliger* (Kieffer), *D. tristriatus* Kieffer, *Iphiaulax* sp., *Parallorhogas pallidiceps* Perkins, *Promiscolus sesquistriatus* Kieffer, *Pristodoryctes striativentris* Kieffer and *Rinamba opacicollis* Cameron	[20,38,93,[96], [97], [98]]
4	Encyrtidae	*Avetinella* sp.	[38]
5	Eupelmidae	*Metapelma* sp.	[62]
6	Eurytomidae	*Eurytoma* sp., *Eurytoma xylotrchi* Ferriere	[38,99]
7	Gasteruptiidae	*Gasteruption* sp.	[97]
8	Ichneumonidae	*Epixorides caerulescens* (Morley), *Paraglypta tuigera* (Kieffer)	[20,100,101]
9	Stephanidae	*Diastephanus* sp.	[101]
**Predatory birds**			
10	Megalaimidae	*Megalaima* sp., *Megalaima viridis* Boddaert	[62,102]
11	Picidae	Woodpecker	[70]
**Microbial Bioagents**			
12	Trichocomaceae	*Aspergillus tamarii* Kita	[97]
14	Cordycipitaceae	*Beauveria bassiana* (Bals-Criv.) Vuill	[[103], [104], [105]]
15	Clavicipitaceae	*Metarhizium anisopliae* (Metchnikoff) Sorokin	[[15], [105]]
**Rhabditida (Nematodes)**			
11	Steinernematidae	*Steinernema carpocapsae* (Weiser)	[105]
12	Heterorhabditidae	*Heterorhabditis* sp.	

The ochratoxin produced by *Aspergillus* and *Penicillium* species, is more dangerous to borers and can be used to develop novel pesticides [13,92]. The *B. bassiana* strains with 2 × 10^8^ CFU can cause 43.33–48.89% mortality in the field condition [21], but the same organism caused 28% mortality in Hawaii fields [106]. In contrast, *B. bassiana* caused 90% mortality in laboratory conditions in China [56] and 100% mortality in Indian field conditions [88], indicating the importance of local climate in biocontrol agent effectiveness.

The biological formulations *viz., Bacillus subtilis* @ 10/l and azadirachtin 1% EC @ 1ml/lit are used against borer infestation. The neem leaves paste must be pasted two times a year during March and September. Neem seed kernel extract can be sprayed on the main stem of the coffee plant [87,105]. Some of the important botanicals like *Allium sativum* L., *Allium cepa* L., *Azadirachta indica* A. Juss., *Eupatorium adenophorum* Spreng., *Utrica dioca* L. and *Artemesia indica* Willd are also used by farmers for managing the CWSB [58]. So, a study can be conducted on the strain improvement of various microbes and their toxin for developing ecofriendly pesticide. Use of microbial products and different botanicals mixtures may increase the efficacy against borer as they can be used as a tool in IPM packages.

### 6.6 Chemical

A chemical method is used as a last and final method to control the borer. Stem injection or root feeding of the recommended insecticides, as well as stem coating with sealer cum healer, proved to be the most effective means of controlling borer infestations. Spraying or coating the coffee main stem with 10% lime before the adult flight reduces egg laying by the adults [85,105]. Insecticides such as BHC, lindane, aldrin and dieldrin are very effective and have demonstrated excellent control, but their use is prohibited because they are non-selective and have a negative impact on the environment [86,107].

Insecticides such as chlorpyrifos, fipronil and imidacloprid, combined with a 10% lime solution, have been shown to be effective in borer management [86]. Some of the most commonly used insecticides against borers include chlorpyrifos 50 EC + cypermethrin 5 EC @ 1.2 ml/l [84,105], chlorantraniliprole 20 SC @ 0.5 ml/l, chlorpyrifos 20 EC @ 3 ml/l, and fipronil 40% + imidacloprid 40% WG @ 0.5 g/l [21] can control damage caused by the borer.

To protect the healthy plants, combining cultural methods *viz.,* wrapping the main stem and thick primary branch with a gunny bag, followed by spraying insecticides like chlorpyrifos 50 EC + cypermethrin 5 EC @ 1.2 ml/l, reduces the pest to a greater extent [84,105,108]. Swabbing the main stem and thick primaries [107] with 10% lime solution (20 kg spray lime dissolved in 200 l of water along with 200 g of DDL fevicol) by the end of March prevents egg-laying and a similar swabbing process with insecticide, phenthoate 50 EC @ 400 ml per 200 L of water along with 200 ml of any wetting agent before mid-April could effectively control CWSB [89]. Most of the insecticides using in the coffee ecosystems are broad spectrum outdated and banned or at verge of banning which are causing severe impact on the ecosystem. Hence, study must be initiated by using the new age chemicals with different combinations to manage the pest effectively.

## 7 Conclusion

The pest is historical and documented a century before from the coffee-growing region and causing considerable damage. Several techniques followed to manage the pest from past 100 years, but it is still insufficient to combat this pest. The failure is mainly due to the lack of ground information on the ecological and behavioural adoption of the borer. Practicing different methods of management without having strong knowledge of the pest behaviour leads to greater failure. The details on the biology, host selection, beahvioural and chemical ecology of the pest will show a new avenue for pest management by identifying tolerant cultivars, biopesticides and developing specific pheromones to deceive or trap the adults. Among multiple control methods, use of single tool to manage this pest is not efficient and economical. Hence, combined/harmonious use of different tools would reduce the pest load in the plantation and provide increased yield. So, studies needs to be conducted on the scanty area of the coffee white stem borer to understand the pest in detail and to formulate the precise management techniques is advisable.

## Funding

2018YFD0201102-4.

## Author contribution statement

All authors listed have significantly contributed to the development and the writing of this article.

## Data availability statement

No data was used for the research described in the article.

## Additional information

No additional information is available for this paper.

## Declaration of competing interest

The authors declare that they have no known competing financial interests or personal relationships that could have appeared to influence the work reported in this paper.

## References

[1] Dastagiri M.B. (2017). Analysis of economic trends in overseas markets for Indian tea and coffee. Outlook Agric..

[2] The Coffee Board of India (2020).

[3] Lee H.L., Lee C.Y. (2007).

[4] The Coffee Board of India (2019).

[5] Garcia C.A., Bhagwat S.A., Ghazoul J., Nath C.D., Nanaya K.M., Kushalappa C.G. (2010). Biodiversity conservation in agricultural landscapes: challenges and opportunities of coffee agroforests in the Western Ghats, India. Conserv. Biol..

[6] Rajus S., Bhagavan S.G., Kharva H., Rao S., Olsson S.B. (2021). Behavioral ecology of the coffee white stem borer: toward ecology-based pest management of India’s coffee plantations. Front. Ecol. Evol..

[7] Waller J.M., Bigger M., Hillocks R.J., Waller J.M., Bigger M., Hillocks R.J. (2007). Coffee Pests, Diseases and Their Management.

[8] Jonsson M., Raphael I.A., Ekbom B., Kyamanywa S., Karungi J. (2014). Contrasting effects of shade level and altitude on two important coffee pests. J. Pest. Sci..

[9] Giddegowda V.M., Dinesh A.S. (2012). The coffee white stemborer *Xylotrechus quadripes* (Coleoptera: Cerambycidae): bioecology, status and management. Int. J. Trop. Insect Sci..

[10] Thapa S., Lantinga E.A. (2016). Infestation by coffee white stem borer, *Xylotrechus quadripes*, in relation to soil and plant nutrient content and associated quality aspects. Southwest. Entomol..

[11] Vega F.E., Posada F., Francisco I. (2006). Encyc Pest Manag.

[12] Egonyu J.P., Kucel P., Kagezi G. (2015). *Coffea arabica* variety KP423 may be resistant to the Cerambycid coffee stemborer *Monochamus leuconotus*, but common stem treatments seem ineffective against the pest. Afr. Entomol..

[13] Gichuhi J.M. (2013).

[14] Venkatesha M.G., Dinesh A.S. (2012). The coffee white stemborer *Xylotrechus quadripes* (Coleoptera: Cerambycidae): bioecology, status and management. Int. J. Trop. Insect Sci..

[15] Karanja L., Phiri W., Odour N.A., G I (2012).

[16] Murphy T.S., Phiri N.A., Sreedharan K., Kutywayo D., Chanika C. (2008).

[17] Liebig T., Babin R., Ribeyre F., Laderach P., Asten P., Poehling H.M., Jassogne L., Cilas C., Avelino J. (2018). Local and regional drivers of the African coffee white stem borer (*Monochamus leuconotus*) in Uganda. Agric. For. Entomol..

[18] Kariyanna B., Sunitha N.D., Bheemanna M., Drumont A., Vitali F., Kurzawa J. (2022).

[19] Dauber D., Hawkeswood T.J. (1993).

[20] Le Pelley R.H. (1968).

[21] Manikandan K.R., Muthuswami M., Chitra N., Ananthan M. (2019). Management of coffee white stem borer *Xylotrechus quadripes* (Chevrolat, 1863) Coleoptera: Cerambycidae) in the lower pulney hills, India. Int. J. Curr. Microbiol. App. Sci..

[22] Kariyanna B., Mohan M., Gupta R., Vitali F. (2017). The checklist of longhorn beetles (Coleoptera: Cerambycidae) from India (Monograph). Zootaxa.

[23] Gahan C.J. (1906). Coleoptera: Cerambycidae.

[24] Stokes H. (1838).

[25] Hayashi M., Makihara H. (1981). The Cerambycidae (Coleoptera) of Nepal collected by the Kyushu University scientific expedition. Esakia.

[26] Chien T.Y. (1989). Records of the larvae of seven species of *Xylotrechus* from China (Coleoptera: Cerambycidae). Acta Entomol. Serbica.

[27] Visitpanich J. (1994). The biology and survival rate of the coffee stemborer, *Xylotrechus quadripes* Chevrolat (Coleoptera, Cerambycidae) in Northern Thailand. Jpn. J. Entomol..

[28] Rhainds M., Warndorff M., PhangKok C., ChinChiew L., Gries G. (2001). Stimuli increasing oviposition by female coffee white stemborer (Coleoptera: Cerambycidae). Can. Entomol..

[29] YouSheng Z., ZhongXi Z., SongLin L., DingAn L., QingHui Z., Hua W. (2002). Ecology and occurrence pattern of *Xylotrechus quadripes* Chevrolat and its integrated control. J. Southwest Agri. Uni..

[30] Kariyanna B., Gupta R., Bakthavatchalam N., Mohan M., Nithish A., Dinkar N.K. (2017). Host plants record and distribution status of agriculturally important longhorn beetles (Coleoptera: Cerambycidae) from India. Progressive Research.

[31] Kariyanna B. (2016). Department of Entomology, College of Agriculture, Raipur, Faculty of Agriculture, Indira Gandhi Krishi Vishvavidyalaya.

[32] Hanks L.M. (1999). Influence of the larval host plant on reproductive strategies of cerambycid beetles. Annu. Rev. Entomol..

[33] Schoeman P.S., Hamburg H., Pasques B.P. (1998). The morphology and phenology of the coffee white stem borer, *Monochamus leuconotus* (Pascoe) (Coleoptera: Cerambycidae), a pest of Arabica coffee. Afr. Entomol..

[34] Venkatesha M.G., Seetharama H.G. (1999). Sexing of adults of coffee white stemborer, *Xylotrechus quadripes* (Chevr.) (Coleoptera: Cerambycidae). Entomon.

[35] Jayarama M.G. Venkatesha, Souza M.V., Seetharama H.G., Srinivasan C.S., Naidu R., Hall D.R., Cork A., Muraleedharan M., Rajkumar R.E. (2000). Recent Advances in Plantation Crops Research.

[36] Joshi A.R. (2018). https://www.chinawaterrisk.org/opinions/can-nepali-coffee-survive-the-impacts-of-climate-change/.

[37] Pandey M., Kayastha P., Khanal S., Shrestha S., Thakur G., Adhikari K., Shah K.K., Pant D., Khanal D. (2022). An Overview on Possible Management Strategies for Coffee White Stem Borer *Xylotrechus quadripes* Chevrolat (Coleoptera: Cerambycidae) in Nepal. Heliyon.

[38] Shylesha A.N., Veeresh G.K., Sidde Gowda D.K. (1992). New record of egg parasite and survey of natural enemies complex of white stem borer of coffee, *Xylotrechus quadriceps* Chevr. J. Plant. Crops.

[39] Shruthy G.M.K. (2016). White Stem Borer (WSB) in Western Ghats and shifts towards Robusta coffee: evidences from a recent household survey of Arabica coffee growers in Kodagu district of Karnataka state. Agri. Situat. India.

[40] CCRI (2003).

[41] Duffy E.A.J. (1968).

[42] Santosh P., Sreenath H.L., Kumar P.K.V., Seetharama H.G. (2011). Jayarama. New record of coffee white stemborer *Xylotrechus quadripes* Chevrolat on *Psilanthus bengalensis* in India. J. Plant. Crops.

[43] Veeresh G.K. (1995).

[44] Kariyanna B., Mohan M., Das U., Biradar R., Anusha Hugar A. (2017). Important longhorn beetles (Coleoptera: Cerambycidae) of horticulture crops. J. Entomol. Zool. Stud..

[45] Rhainds M., Warndorff, Chiew P.K., Lan C.C., Gries G. (2001). Stimuli increasing oviposition by female coffee white stem borer (Coleoptera: Cerambycidae). Can. Entomol..

[46] Jaramillo J., Muchugu E., Vega F.E., Davis A., Borgemesister C. (2011). Some like it hot: the influence and implications of climate change on coffee berry borer (*Hypothenemus hampei*) and coffee production in East Africa. PLoS One.

[47] Gillison A.N., Liswanti N., Budidarso S., Noordwijk M.V., Tomich T.P. (2004). Impact of cropping methods on biodiversity in coffee agroecosystems in Sumatra, Indonesia. Ecol. Soc..

[48] African Development Bank Group (2010).

[49] Hall D.R., Cork A., Phythian S.J., Chittamuru S., Jayarama B.K., Venkatesha M.G., Sreedharan K., Kumar P.K.V., Seetharama H.G., Naidu R. (2006). Identification of components of male-produced pheromone of coffee white stem borer, *Xylotrechus quadripes*. J. Chem. Ecol..

[50] Radhakrishnan S., Ramaiah P.K., Bhat P.K. (1987). Methodology to estimate yield loss in coffee due to insect pests. J. Coffee Res..

[51] Naidu R. (1997). White stem borer in coffee, current management and future strategies. Planters Chronicle.

[52] Venkatesha M.G. (2010). Proceedings of the 16th Asian Agricultural Symposium and 1^st^ International Symposium on Agricultural Technology.

[53] Kuang B.Q. (1959). A primary investigation on the coffee white stem borrer, *Xylotrechus quadripes* Chev. Insect Knowledge.

[54] Kumar A.R., Gopinandhan T.N., Reddy P.K., Uma M.S., Patil S., Reddy G.V.M., Seetharama H.G. (2019). Assessment of crop loss in Arabica coffee due to white stem borer, *Xylotrechus quadripes* Chevrolat (Coleoptera: Cerambycidae) infestation. J. Plant. Crops.

[55] Jayaraj J., Muthukrishnan N. (2013). Management of white stem borer in coffee. The Hindu.

[56] Wei J., Kuang R. (2000). Application of capture-recapture models for estimating coffee stemborer (Coleoptera: Cerambycidae) abundance. Int. J. Trop. Insect Sci..

[57] NARC (2007). Nepal SIMI annual performance report.

[58] Panthi B.B. (2014). Small scale coffee farmer’s response towards management of coffee pest through field level techniques. J. Indian Inst. Sci..

[59] Knight C.D. (1939). Observations on the life-history and control of the white borer of coffee in Kenya. East Afr. agric..

[60] Seetharama H.G., Vasudev V., Kumar P.K.V., Sreedharan K. (2004). Studies on the biology of coffee stemborer–a method to facilitate oviposition in the laboratory. J. Coffee Res..

[61] Pattan F.R., Khan H.K. (2017). Intermittent mating and egg laying by coffee white stem borer, *Xylotrechus quadripes* Chevrolat (Coleoptera: Cerambycidae). Mysore J. Agric. Sci..

[62] Subramaniam T.V. (1934).

[63] Seetharama H.G., Vasudev V., Kumar P.K.V., Sreedharan K. (2005). Biology of coffee white stemborer *Xylotrechus quadripes* Chev. (Coleoptera: Cerambycidae). J. Coffee Res..

[64] Logan W.J.C. (1987).

[65] Shoeman P.S., Hamburg H.V., Pasques B.P. (1998). The morphology and phenology of the coffee white stem borer, *Monochamus leuconotus* (Pascoe) (Coleoptera: Cerambycidae), a pest of Arabica coffee. Afr. Entomol..

[66] Kariyanna B., Gupta R., Mohan M., Vitali F. (2019). Wood-boring longhorn beetles (Cerambycidae: Coleoptera) of agro-forest ecosystem in India. Indian J. Entomol..

[67] Sekhar P.S. (1958). Pests of coffee and their control. Indian Coffee.

[68] Kung P.C. (1977). Studies on two long-horned beetles infesting coffee trees in Kwangsi Autonomous Region. Acta Entomol. Serbica.

[69] Linsley E.G. (1959). Ecology of Cerambycidae. Annu. Rev. Entomol..

[70] Tapley R.G. (1960). The white coffee borer, Anthores leuconotus Pasc. and its control. Bull. Entomol. Res..

[71] Venkatesha M.G., Bhat P.K., Seetharama H.G. (1995). Courtship behaviour of the coffee white stem borer, *Xylotrechus quadripes* (Chevr.) (Coleoptera: Cerambycidae). J. Coffee Res..

[72] Seetharama H.G., Vasudev V., Kumar P.K.V., Sreedharan K. (2004). Behaviour of coffee white stem borer beetle in the field. J. Coffee Res..

[73] Pang J.X., Zeng X., Zhu J.Y., Liu N.Y. (2018). Chemosensory transmembrane protein families in the coffee white stemborer, *Xylotrechus quadripes* (Coleoptera: Cerambycidae). Environ. Entomol..

[74] Gowda D.K.S., Veeresh G.K., Krishnamoorthy P., Venkatesha M.G. (1991). Tunneling behaviour of white stem-borer *Xylotrechus quadripes* Chevr. (Coleoptera:Cerambycidae). J. Coffee Res..

[75] Iwabuchi K. (1987). Mating behaviour of *Xylotrechus pyrrhoderus* Bates (Coleoptera: Cerambycidae) female mounting behaviour. Appl. Entomol. Zool..

[76] (2014). Prashant Studies on Chemical Ecology and Behaviour of Coffee White Stem Borer (CWSB), *Xylotrechus quadripes* Chevrolat (Coleoptera: Cerambycidae).

[77] Yin Y.J., Zhao, Zhu J.Y., Liu N.Y. (2019). Antennal UDP-glycosyltransferase genes in the coffee white stemborer, *Xylotrechus quadripes*. J. Asia Pac. Entomol..

[78] Conchou L., Lucas P., Meslin C., Proffit M., Staudt M., Renou M. (2019). Insect odorscapes: from plant volatiles to natural olfactory scenes. Front. Physiol..

[79] Luo Z., Chen Y., Liu L., Pan G., Wang R. (2021). Analysis of volatile components in host plants of *Xylotrechus quadripes*. The fourth international workshop on environment and geoscience. IOP Conf. Ser. Earth Environ. Sci..

[80] Reddy G.M., Venkateshalu, Swamy H.M., Asokan R., Reddy P.V.R. (2022). Genetic characterization and DNA barcoding of the coffee white stem borer, *Xylotrechus quadripes* Chevrolat (Coleoptera: Cerambycidae) infesting Robusta coffee (*Coffea canephora*). Pest Manag. Hortic. Ecosyst..

[81] Prakash N.S., Suresh Kumar V.B., Chethan J., Das Divya K., Seetharama H.G., Roobak Kumar A., Uma M.S., Krishna Reddy P., Raghuramulu Y. (2020). Breeding for coffee white stem borer tolerance – significant breakthrough. India Cofee, the coffe magazine.

[82] Aryal L., Basnet S., Aryal S. (2022). Field screening of arabica coffee genotypes against coffee white stem borer (*Xylotrechus quadripes*) and leaf rust (*Hemileia vastatrix*) infestation in kaski, Nepal. J. Agri. Environ..

[83] Santosh P., Bharathi K., Sreenath H. (2023). Identification of defense related transcripts in robusta coffee (*Coffea canephora*) in response to infestation by coffee white stem borer (*Xylotrechus quadripes*) using forward subtracted suppression subtractive hybridization library. Research Square.

[84] Uma M.S., Seetharama H.G., Reddy G.M., Kumar T.M., Ashwitha T.V., Raghuramalu Y. (2017). A novel approach to arrest white stem borer in coffee. J. Exp. Zool..

[85] Rajbhandari R., Das, Thapa R.B. (2015). http://www.plantwise.org/KnowledgeBank/pmdg/20167800006.

[86] Rutherford A., Phiri N. (2006).

[87] Rajbhandari R.D. (2014). https://www.plantwise.org/FullTextPDF/2014/20147801455.pdf.

[88] CCRI (2017).

[89] CCRI (2022).

[90] Mangalgikar P., Bhanu K.R.M., Belavadi V., Kumar K.V., Muniyappa C., Ammagarahalli B., 1-octadecene (2023). A female produced aggregation pheromone of the coffee white stem borer (*Xylotrechus quadripes*). Horticulturae.

[91] NTCDB (2017). https://www.teacoffee.gov.np/public/images/pdf-215463343.41.

[92] Zha C., Cohen A.C. (2014). Effects of anti-fungal compounds on feeding behavior and nutritional ecology of tobacco budworm and painted lady butterfly larvae. Entomol., Ornithol. Herpetol..

[93] Venkatesha M., Seetharama H., Sreedharan K. (1997). *Iphiaulax* sp.–a new braconid parasitoid of coffee white stemborer, *Xylotrechus quadripes* (Chevr.)(Coleoptera: Cerambycidae). Pest Manag. Hortic. Ecosyst..

[94] Azevedo C.O., Waichert C. (2006). A new species of *Apenesia* (Hymenoptera, Bethylidae) from India, a parasitoid of coffee white stem borer *Xylotrechus quadripes* (Coleoptera, Cerambycidae). Zootaxa.

[95] Kieffer J.J. (1921). On various Hymenoptera destroying cerambycids visible to coffee and bamboo. Bull. Agric. Inst. Sci..

[96] Prakasan C.B., Sreedharan K., Bhat P. (1986). New record of a parasite of coffee white stem-borer *Xylotrechus quadripes* Chevr. from India. J. Coffee Res..

[97] CCRI (1998).

[98] Roobakkumar A., Seetharama H., Reddy P.K., Uma M., Ranjith A.P. (2020). First report of *Rinamba opacicollis* cameron (Hymenoptera: Braconidae, Doryctinae) in India as parasitoid of coffee stem borer, *Xylotrechus quadripes* (Chevrolat) (Coleoptera: Cerambycidae). Entomon.

[99] Ferriere C. (1933). Description of a parasitic chalcidian of longhorn beetle in Indo-China. Bull. Entomol. Soc. France.

[100] Thompson W.R. (1947). A catalogue of the parasites and predators of insect pests. Commonw. Inst. Biol. Control.

[101] Visitpanich J. (1994). The parasitoid wasps of the coffee stem borer, *Xylotrechus quadripes* Chevrolat (Coleoptera, Cerambycidae) in Northern Thailand. Jpn. J. Entomol..

[102] Yahya H.S.A. (1982). Observations on the feeding behaviour of barbet (*Megalaima* sp.) in coffee estates of South India. J. Coffee Res..

[103] Balakrishnan M.M., Sreedharan K., Bhat P.K. (1994). Occurrence of the entomopathogenic fungus *Beauveria bassiana* on certain coffee pests in India. J. Coffee Res..

[104] Jia-ning W., Rong-pid K. (2002). Biological control of coffee stem borers. Entomol. Sin..

[105] Reddy K.P., Uma M.S., Roobak Kumar A., Seetharama H.G., Rao S.P., Y (2020). Raghuramulu. Research carried out on CWSB – an over view. India Cofee, the coffe magazine.

[106] Aristizabal A.L.F., Baker P.S., Orozco H.J., Chaves C.B. (1997). Parasitism of *Cephalonomia stephanoderis* Betrem on population of *Hypothenemus hampei* Ferrari at infestation low levels in the field. Rev. Colomb. Entomol..

[107] Roobak Kumar A., Uma M.S., Krishna Reddy P., Seetharama H.G., Rao S.P., Raghuramulu Y. (2020). Coffee white stem borer - a saga of 100 years of Research. India Cofee, the coffe magazine.

[108] Reddy K., Kumar R., Reddy M., Seetharama H.G., Dhanam M. (2019). Evaluation of insecticides against white stem borer, *Xylotrechus quadripes* (Cerambycidae: Coleoptera) infesting coffee. J. Entomol. Zool. Stud..

